# Relationships Among Structures, Team Processes, and Outcomes for Service Users in Quebec Mental Health Service Networks

**DOI:** 10.5334/ijic.4718

**Published:** 2020-06-11

**Authors:** Marie-Josée Fleury, Guy Grenier, Jean-Marie Bamvita

**Affiliations:** 1 Department of Psychiatry, McGill University, Douglas Hospital Research Centre, Montreal, Quebec, CA; 2 Douglas Hospital Research Centre, Montreal, Quebec, CA

**Keywords:** mental health reform, cluster analysis, mental health service networks, team processes, services user outcomes

## Abstract

**Background:**

Few studies have identified and compared profiles of mental health service networks (MHSN) in terms of structures, processes, and outcomes, based on cluster analyses and perceptions of team managers, MH professionals and service users. This study assessed these associations in Quebec metropolitan, urban and semi-urban MHSN.

**Methods:**

A framework adapted from the Donabedian model guided data management, and cluster analyses were used to identify categories. Study participants included team managers (n = 45), MH professionals (n = 311) and service users (n = 327).

**Results:**

For all three MHSN, a common outcome category emerged: service users with complex MH problems and negative outcomes. The Metropolitan network reported two categories for structures (specialized MH teams, primary care MH teams) and processes (senior medical professional, psychosocial professionals), and outcomes (middle-age men with positive outcomes, older women with few MH problems). The Urban and Semi-urban networks revealed one category for structures (all teams) and service user (young service users with drug disorders), but two for processes (psychosocial professionals: urban, all professionals: semi-urban).

**Conclusion:**

The Metropolitan MHSN showed greater heterogeneity regarding structures and team processes than the other two MHSN. Service user outcomes were largely associated with clinical characteristics, regardless of network configurations for structures and team processes.

## Introduction

Integrated care models are increasingly implemented internationally to reduce healthcare service fragmentation and better meet the needs of patients [1,2,3]. Person-centered and integrated care are also recognized for their positive impact on service providers by reducing hospital readmission or costs, as well as for boosting job satisfaction among health care professionals and improving perceived health and satisfaction with services among service users [2,4,5]. Ongoing mental health (MH) reforms since the early 2000s have aimed particularly at improving integrated care and increasing service quality [6]. Following international trends and the 2005 Quebec MH Action Plan [7], health and social service centers (HSCC) were created from mergers of general hospitals, nursing homes and local community service centers (LCSC) [8]. HSCC were responsible for integrating MH services within their respective MH service networks (MHSN), bringing together specialized MH services and new MH primary care teams, introducing integration strategies including service agreements and deploying liaison officers.

The development of a theoretical framework for assessing the implementation of integrated care has been the subject of various studies [9]. The Donebedian model [10] is one of the main models used for assessing the quality of health services [11,12] and partnerships [11]. The Donabedian model is based on the theory that structures bear on team processes, and in turn, on health service outcomes [11]. MHSN with stronger structural elements such as greater financial resources or levels of service integration are expected to produce better outcomes for service users [13]; while effective teamwork among multidisciplinary professionals should promote high quality service delivery [14,15], and improve satisfaction among service users [16]. According to a recent literature review, team processes are the cornerstone of integrated care [9]. Team effectiveness is associated with processes such as work role performance (i.e. actions and behaviors of team members involved in executing tasks) [17,18], professional autonomy [19], team collaboration [20], and decision-making [21]. A recent study found that combinations of different organizational cultures were associated with higher levels of perceived integrated care among patients [22]. While some studies have identified factors related to structures, processes and outcomes in explaining the overall success or failure of integrated care [9,23,24,25], few studies to our knowledge have used cluster analysis to identify and compare profiles of MHSN in terms of structures, processes, and outcomes, based on the perceptions of team managers, MH professionals (MHP) and service users. Local-level reforms have important implications for MHP [26]. Teams bring together professionals with different values, experiences and practices, increasing the risk of interpersonal conflict [27] and reducing professional autonomy somewhat [28,29]. It is also reported than 50–70% of inter-organizational collaboration fails [25]. Moreover, the transformation of health systems to provide person-centered and integrated care continues to represent a challenge for managers and professionals alike [16]. While MH reforms have focused on meeting the needs of service users, promoting recovery and quality of life (QOL), few studies [30,31,32] have linked these outcomes to the organization of services (structures) in various territories, or to professional and team characteristics (processes).

A reliable method for identifying categories based on groupings of different types of variables associated with subjects is cluster analysis [33]. Cluster analysis has often been used for the creation of typologies of MH service users based on their sociodemographic, clinical and service use characteristics [34,35,36,37,38]. However, to our knowledge, no previous study has used cluster analysis to identify categories that take into account particular characteristics of MH structures, MH teams and MH services users in networks.

This study aimed to understand how different configurations of MHSN and teams impact on service user outcomes, by identifying through cluster analyses specific categories of associated variables within three Quebec MHSN, based on interrelationships among: 1) MH settings, including territorial and organizational features (structures); 2) characteristics of MHP, including team process variables (e.g., team support, team autonomy); and 3) service user characteristics, including socio-demographic variables, clinical characteristics, and outcomes (e.g., QOL, recovery). Based on the Donabedian model, we hypothesized that more positive structures would relate to better team processes in MHSN and in turn to more positive outcomes for service users.

## Methods

### Setting

This study was conducted in three Quebec MHSN selected for type of territory (urban, semi-urban), population demographics, diversity of services offered, implementation of integration strategies, and use of evidence-based practices. Network 1 was labelled “Metropolitan MHSN”, Network 2, “Urban MHSN” and Network 3 “Semi-urban MHSN”. Table 1 shows the main characteristics of the three MHSN.

**Table 1:** Description of the three Mental Health (MH) Service Networks.

Networks	Network 1	Network 2	Network 3

Label	*Metropolitan MH service network*	*Urban MH service network*	*Semi-urban MH service network*
Area	Metropolitan and university	Urban and university	Remote
Population	374, 655	311,455	75,807
Proportion of population with low income	21.5%	4.5%	10.0%
Proportion of single parent families	38%	19%	21%
Adjusted average suicide mortality rate by 100 000 inhabitants	14.9	20.4	16.0
Government financial support for MH per inhabitant	210.74 CAN$	207.67 CAN$	125.76 CAN$
Percentage of financial support for MH community organisations	7.0%	8.9%	11.0%
Primary care services	HSCC^a^ (n = 2) LCSC^b^ (n = 6) Medical clinics (n = 16) Adult primary care teams (n = 2)	HSCC (n = 1) LCSC (n = 7) Medical clinics (n = 59) Adult primary care teams (n = 3)	HSCC (n = 1) LCSC (n = 1) Medical clinics (n = 10) Adult primary care teams (n = 1)
Number of full-time professionals in MH primary care teams	52	101	15
Number of general practitioners	240	456	106
MH community based organisations	30	40	12
Psychologist for 10 000 inhabitants	15.23	16.56	10.14
MH specialized services	MH university Institute-psychiatric ER^c^ (n = 1) Short-term care inpatient units (102 beds) Day hospitals (n = 3) Outpatient clinic Assertive community treatment Intensive case management Specialized clinics (n = 5) Psychosocial rehabilitation program	MH university institute- psychiatric ER (n = 3) Short-term care inpatient units (128 beds) Day hospital (n = 1) Outpatient clinic Assertive community treatment Intensive case management Specialized clinics (n = 3) Treatment centres in the community (n = 3)	General hospital-general ER (n = 1) Short-term care inpatient units (27 beds) Day hospital (n = 1) Outpatient clinic Assertive community treatment Intensive case management

^a^ Health and social service center. ^b^ Local community service center. ^c^ Emergency room.

### Sample and data collection

Data came from four sources: 1) documentation from organizations and MH teams in each selected network; and questionnaires completed by 2) team managers, 3) MHP, and 4) service users. Documents consulted between November 2012 and March 2013 provided data on population demographics, government financial support for MH, and service provider characteristics. Questionnaires were completed as follows: team managers (October 2013 to June 2014), MHP (May to November 2013), and service users (June 2013 to August 2014).

An advisory committee consisting of key decision makers from the MHSN was established to help with recruitment, validate instruments, and support data collection. The committee identified all MH managers from the three networks, who in turn identified professionals from network MH teams. Regarding eligibility, MHP and managers had to be working on MH teams with at least three professionals representing two or more disciplines, in public MH organizations. These criteria drew upon previous research on teamwork, while accounting for the intensity and multidisciplinary nature of professional involvement in complex MH cases [39].

Team managers and MHP were invited to the study by email or telephone. The self-administered questionnaires took about 60 minutes to complete. Service users were recruited through posters displayed at HSSCs or hospitals, or were referred by MHP. Participants had to be 18–70 years old with a DSM-V diagnosis. Those unable to participate and provide informed consent due to clinical instability, intellectual impairment, hospitalization, or involuntary treatment order, were excluded. Two 90-minute interviews were conducted with each participating service user at one-week intervals. The research ethics board of a MH university institute approved the study protocol. All participants signed a consent form.

Of 49 managers recruited, 45 participated for a 92% response rate. Mean age was 44, and 71% (n = 32) were female; 63% (n = 28) worked in specialized MH services and the others in primary care. No significant differences between respondent and non-respondent managers were found on gender (Pearson Chi-square = .966; df = 1; Fisher’s Exact Test 2-sided P = .663) or team type (Pearson Chi-square = 1.861; df = 2; Fisher’s Exact Test 2-sided P = .245).

Of 466 MHP recruited, 311 participated (67% response), including 108 (35%) medical professionals, 169 (54%) psychosocial professionals, and 34 (11%) general staff. Most professionals (n = 211; 68%) worked in specialized MH services, and were female (n = 218; 70%); mean age was 43. Average seniority in profession was 9 years. There were no significant differences between respondent and non-respondent professionals on the distributions for gender [χ2 (1, N = 466) = 0.03; p = 0.87] or team type [χ2 (1, N = 466) = 0.79; p = 0.68].

Of 389 MH service users recruited, 327 participated (84% response). Their average age was 48, and gender was equally divided (male: n = 163; female: n = 164). Service users had 1.8 MH disorders on average, most commonly mood disorders (n = 144; 44%) and schizophrenia (n = 97; 30%). No significant differences emerged between respondents and non-respondents on age (ANOVA t test: F = 620; P = 0.453) or gender (Pearson Chi-Square = 0.522; P = 0.829).

### Conceptual framework and instruments

**Figure 1:**
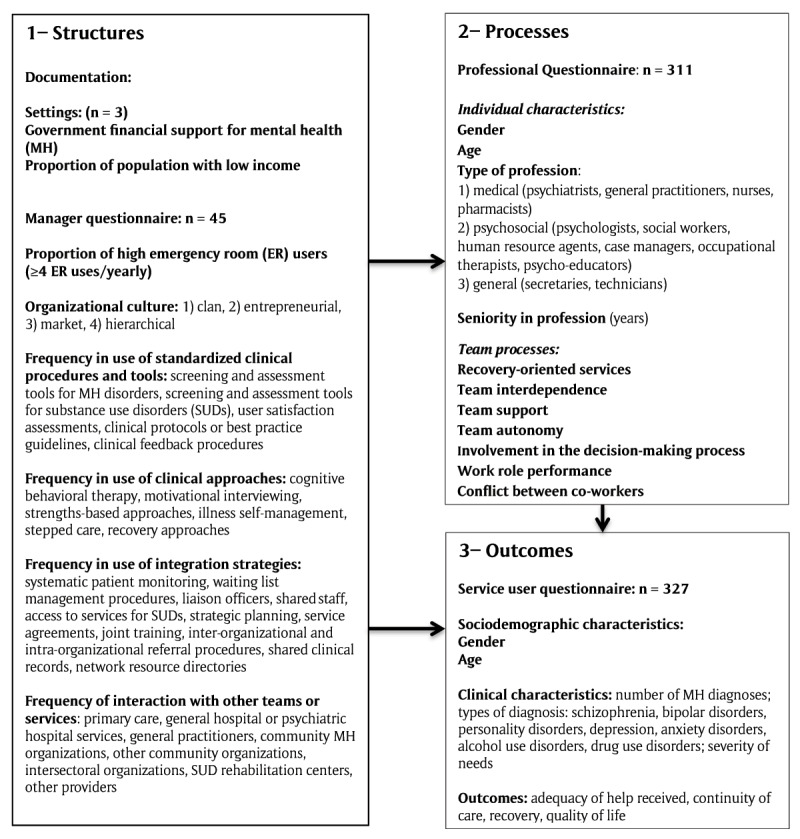
Conceptual Framework.

The conceptual framework adapted from the Donabedian model guided data collection and analysis (Figure 1). Regarding structures, variables extracted from documentation included government financial support for MH, and proportions of network inhabitants with low income. The manager questionnaire provided data on high emergency room (ER) users (≥4 visits/yearly) [40]; use of standardized clinical procedures and tools, clinical approaches, integration strategies, and interactions with other teams or services, with all variables measured on five-point Likert scales and calculated as global scores. An additional variable, organizational culture, was assessed with the Organizational Culture Assessment Instrument (OCAI) [41]. This measure consists of six questions, with responses based on distributions of 100 points among four possible choices. A four-factor model for organizational culture was developed along two axes (flexibility vs. stability; internal focus vs. external focus), resulting in four cultural types: 1) clan/family (flexibility; internal focus); 2) adhocracy/entrepreneurial (flexibility; external focus); 3) market/rational (stability; external focus); and 4) hierarchical (stability, internal focus) [42].

Regarding processes, variables from the professional questionnaire included individual characteristics (e.g. age, type of profession) and team processes. Team processes are mechanisms enabling or hindering the capacity for teamwork [43]. They include recovery-oriented services, team interdependence, team support, team autonomy, involvement in the decision-making process, work role performance, conflict between co-workers, team collaboration, and job satisfaction. All team processes were assessed with standardized tools, using seven-point Likert scales. Standardized tools were translated into French and validated. Table 2 presents details on the nine standardized tools, including the Cronbach’s alpha coefficients from their original validation and from validation of the French translation.

**Table 2:** Description of standardized instruments (MH professional questionnaire).

Measures and references	Description	Cronbach’s Alpha Coefficients from the original validation	Cronbach’s Alpha Coefficients from the original validation in French and references

Recovery Oriented-Services [44]	32 items; 5 sub-dimensions (life goals, involvement, diversity of treatment options, choice, individually-tailored services); (7 point scale); Higher = more positive	0.76–0.90	N/A
Team Interdependence [45]	20 items; 3 sub-dimensions (task interdependence, resource interdependence, goal interdependence); (7 point scale); Higher = more positive	0.77–0.88	N/A
Team Support [46]	Team support; (7 point scale); Higher = more positive	0.84–0.85	0.85 [47]
Team Autonomy [48]	3 items; (7 point scale); Higher = more positive	0.76	0.67 [49]
Involvement in the Decision-Making Process [48]	3 items; (7 point scale); Higher = more positive	0.88	0.80 [49]
Work Role Performance [17]	18 items; 6 sub-dimensions (proficiency by the individual, proficiency by the team, adaptivity by individual, adaptivity by the team, proactivity by the individual, proactivity by the team; (7 point scale); Higher = more positive	0.67–0.93	0.87–0.94 [47]
Conflict Between Co-Workers [50]	9 items; 3 sub-dimensions (relationships, tasks, processes); (7 point scale); Higher = more negative	0.93–0.94	0.84–0.91 [51]
Team Collaboration [21]	14 items; 4 sub-dimensions (communication, synchronization, explicit coordination, implicit coordination; (7 point scale); Higher = more positive	0.77–0.91	0.77–0.91 [21]
Job Satisfaction [52]	20 items; 5 sub-dimensions (supervision, contingent reward, operating procedures, co-workers, nature of the work); (7 point scale); Higher = more positive	0.60–0,82	N/A

Outcomes assessed by service users included adequacy of help received, continuity of care, recovery, and QOL based on six standardized questionnaires. Table 3 presents these questionnaires, with Cronbach’s alpha coefficients for both the original validations and validation of the French translation. Sociodemographic characteristics (gender, age) were extracted from the service user questionnaire, and clinical characteristics (number of MH disorders, type e.g., schizophrenia, and severity of needs) from service user medical records (with consent).

**Table 3:** Description of standardized instruments (Service user questionnaire).

Measures and references	Description	Cronbach’s Alpha Coefficients from original validation	Cronbach’s Alpha Coefficients from the original validation in French and reference

Drug Abuse Screening Test (DAST-20) [53]	20 items; (2 point scale); Rating: 0 to 20; Higher = more negative	0.92	N/A
Alcohol Use Disorders Identification Test (AUDIT) [54]	10 items; (5 point scale); Rating: 10 to 50; Higher = more negative	0.80	0.87 [55]
Montreal Assessment of Need Questionnaire (MANQ) [56]	Seriousness of needs; 26 items; (11 point scale); Rating: 0 to 260; Higher = more negative	N/A	N/A
	Adequacy of help received [57]; 26 items (quality and quantity); (11 point scale); Rating: 0 to 520; Higher = more positive	0.91	0.91 [56]
Alberta Continuity of Services Scale for Mental Health (ACSS-MH) [58]	43 items; (5 point scale); Rating: 0 to 215; Higher = more positive	0.78-0.92	N/A
Recovery Assessment Scale (RAS) [59]	41 items; (10 point scale); Rating: 0 to 410; Higher = more positive	0.93	0.92 [60]
Satisfaction with Life Domains scale (SLDS) [61]	20 items; (7 point scale); Rating: 0 to 140; Higher = more positive	0.92	0,90 [62]

### Analyses

Data were screened for missing values and outliers; missing values were replaced using multiple imputation methods (<5%). Statistical analyses included univariate and cluster analyses. Univariate analyses consisted of frequency distributions for categorical variables (numbers and percentages), and central tendency distributions for continuous variables (mean values and standard deviations). For the cluster analyses, participant typologies were calculated using two-step cluster analysis with SPSS version 24. The choice of variables for inclusion was driven by their importance based on the Donabedian model [10], and by significant differences in scores between variables for each of the three MHSN. Participant network (i.e. the MHSN to which each participant belonged) was the variable of interest. The Log-likelihood method was used to determine inter-subject distance. A classification of participants was developed using Schwartz Bayesian clustering criteria. The final number of categories of associated variables was set at 4, based on their respective contributions to inter-class homogeneity. Goodness-of-fit was estimated using the measure of cohesion and separation test, and found to be acceptable.

## Results

Table 4 presents a summary of the main results, with a complete description in Table 5.

**Table 4:** Two-step cluster analyses of structures, processes and outcomes in three mental health (MH) service networks. Summary of the main results.

Structures Manager characteristics		Category 1 (n = 10; 22.2%) *“Metropolitan network: primary care teams”*	Category 2 (n = 9; 20.0%) *“Metropolitan network: specialized MH teams”*	Category 3 (n = 19; 42.2%) *“Urban network: all teams”*	Category 4 (n = 7; 15.6%) *“Semi-urban network: allC teams”*

Settings		Metropolitan MH service network	Metropolitan MH service network	Urban MH service network	Semi-urban MH service network ^c^
Government financial support for MH per inhabitant		Highly positive	Highly positive	Positive	Highly negative
Proportion of population with low income		Highly negative	Highly negative	Highly positive	Positive
High emergency room (ER) users		Positive	Highly negative	Positive	Highly positive
Frequency in use of clinical approaches		Highly negative	Positive	Positive	Highly positive
Frequency in use of standardized clinical procedures and tools		Highly negative	Highly positive	Positive	Negative
Organizational culture (Mean, SD)	Clan culture	Highly positive	Highly negative	Negative	Positive
	Entrepreneurial culture	Highly negative	Positive	Negative	Highly positive
	Market culture	Highly negative	Highly positive	Positive	Negative
	Hierarchical culture	Highly negative	Positive	Positive	Highly positive
Frequency of interaction with other teams and services (Mean, SD) (rating: 0–5)		Highly negative	Highly negative	Highly positive	Highly negative
Frequency in use of integration strategies (Mean, SD) (rating: 0–5)		Positive	Highly positive	Highly negative	Negative
**Team processes** **MH professional characteristics**		**Category 1** **(n = 92; 29.6%)** ***“Metropolitan network: psychosocial professionals”***	**Category 2** **(n = 112; 36.0%)** ***Metropolitan and urban networks: senior medical professionals”***	**Category 3** **(n = 69; 22.2%)** ***“Urban network: psychosocial professionals”***	**Category 4** **(n = 38; 12.2%)** ***“Semi-urban network: all professionals”***

Sites		Mostly *Metropolitan MH service network*	Mainly *Metropolitan MH service network*	Exclusively Urban MH service network	Exclusively Semi-urban MH service network
Professions		Mainly psychosocial professions	Mainly medical professions	Exclusively psychosocial professions	Mainly psychosocial professions
Seniority in profession		Medium	Oldest	Old	Youngest
Recovery-Oriented Services		Highly negative	Highly positive	Positive	Negative
Team Interdependence		Highly negative	Highly positive	Negative	Medium
Team Support		Highly negative	Highly positive	Negative	Positive
Team Autonomy		Highly negative	Positive	Medium	Highly positive
Involvement in the Decision–Making Process		Highly negative	Highly positive	Negative	Highly positive
Work Role Performance		Highly negative	Highly positive	Negative	Medium
Conflict Between Co-Workers		Highly negative	Positive	Positive	Highly positive
Team Collaboration		Highly negative	Highly positive	Medium	Positive
Job Satisfaction		Highly negative	Highly positive	Medium	Positive
**Outcomes** **Service user characteristics**		**Category 1** **(n = 84; 25.7%)** ***“Metropolitan network: middle-age men with positive outcomes”***	**Category 2** **(n = 66; 20.1%)** ***“Metropolitan network: older women with few MH problems”***	**Category 3** **(n = 88; 26.9%)** ***“Metropolitan and other networks: service users with complex MH problems and negative outcomes”***	**Category 4** **(n = 89; 27.2%)** ***“Urban and semi-urban networks: young service users with drug disorders”***

Sites		Exclusively Metropolitan MH service network	Exclusively Metropolitan MH service network	From the three networks	Urban and semi-urban MH service networks
Gender		Exclusively male	Exclusively female	Mainly female	Mixed
Age categories		Mainly 45–54	Mainly 55 and over	Mixed	Mainly 18–44
Number of MH disorders		Positive	Highly positive	Highly positive	Positive
Personality disorders		Highly positive	Highly positive	Highly negative	Positive
Drug Abuse Screening Test (DAST)		Positive	Highly positive	Positive	Highly negative
Alcohol Use Disorders Identification Test (AUDIT)		Highly negative	Highly positive	Negative	Positive
Severity of needs		Highly positive	Positive	Highly negative	Negative
Adequacy of help received		Negative	Highly negative	Highly positive	Positive
Alberta Continuity of Services Scale (ACSS) for Mental Health		Highly positive	Negative	Negative	Negative
Recovery Self-Assessment Scale (RSA)		Highly positive	Negative	Highly negative	Medium
Quality of life (Satisfaction with Life Domains Scale – SLDS)		Highly positive	Positive	Highly negative	Negative

**Table 5:** Two-step cluster analyses of structures, processes and outcomes in three mental health (MH) service networks.

Structures Manager characteristics		Category 1 (n = 10; 22.2%) *“Metropolitan network: primary care teams”*		Category 2 (n = 9; 20.0%) *“Metropolitan network: specialized MH teams”*		Category 3 (n = 19; 42.2%) *“Urban network: all teams”*		Category 4 (n = 7; 15.6%) *“Semi-urban network: all teams”*		Combined (n = 45; 10%)	

		n/Mean	%/SD	n/Mean	%/SD	n/Mean	%/SD	n/Mean	%/SD	n/Mean	%/SD

Settings (n., %)	Metropolitan MH service network	10	100%	9	100%	0	0.0%	0	0.0%	19	1000
	Urban MH service network	0	0.0%	0	0.0%	19	100%	0	0.0%	19	100%
	Semi-urban MH service network	0	0.0%	0	0.0%	0	0.0%	7	100.0%	7	100%
Government financial support for MH per inhabitant	125.8 CAN$	0	0.0%	0	0.0%	0	0.0%	7	100.0%	7	100%
	207.7 CAN$	0	0.0%	0	0.0%	19	100%	0	0.0%	19	100
	210.7 CAN$	10	100.%	9	100.%	0	0.0%	0	0.0%	19	100%
Proportion of population with low income (n., %)	21.5%	10	52.6%	9	47.4%	0	0.0%	0	0.0%	19	100%
	10.0%	0	0.0%	0	0.0%	0	0.0%	7	100.0%	7	100%
	<5.0%	0	0.0%	0	0.0%	19	100.0%	0	0.0%	19	100%
High emergency room (ER) users (%, SD)		20.0	27.1	39.3	27.5	27.8	26.4	12.1	15.3	25.9	26.1
Frequency in use of clinical approaches (Mean, SD) (rating: 0–10)		5.5	1.7	6.1	1.8	6.1	1.8	6.4	2.4	6.0	1.9
Frequency in use of standardized clinical procedures and tools (Mean, SD) (rating: 0–42)		24.9	4.3	29.4	5.7	28.4	5.5	25.4	5.5	27.4	5.5
Organizational culture (Mean, SD) (rating: 0–600)	Clan culture	298.8	37.3	163.1	46.5	177.4	63.7	187.7	59.3	203.1	74.5
	Entrepreneurial culture	111.6	31.3	122.2	27.9	113.8	34.5	124.6	53.2	116.7	35.2
	Market culture	66.1	19.3	148.8	21.0	117.9	38.5	89.1	46.0	108.1	43.5
	Hierarchical culture	131.8	33.5	196.0	71.1	198.4	47.0	213.9	87.2	185.5	63.0
Frequency of interaction with other teams and services (Mean, SD) (rating: 0–5)		1.9	0.6	1.9	0.5	2.3	0.8	1.9	0.5	2.1	0,6
Frequency in use of integration strategies (Mean, SD) (rating: 0–5)		3.0	0.6	3.1	0.6	2.6	0.5	2.7	0.6	2.8	0.6
**Team processes** **MH professional characteristics**		**Category 1** **(n = 92; 29.6%)** ***“Metropolitan network: psychosocial professionals”***		**Category 2** **(n = 112; 36.0%)** ***“Metropolitan and urban networks: senior medical professionals”***		**Category 3** **(n = 69; 22.2%)** ***“Urban network: psychosocial professionals”***		**Category 4** **(n = 38; 12.2%)** ***“Semi-urban network: all professionals”***		**Combined** **(n = 311; 100%)**	

		n/Mean	%/SD	n/Mean	%/SD	n/Mean	%/SD	n/Mean	%/SD	n/Mean	%/SD

Sites (n., %)	Metropolitan MH service network	85	92.4%	69	61.6%	0	0.0%	0	0.0%	154	100%
	Urban MH service network	2	2.2%	43	38.4%	69	100%	0	0.0%	114	100%
	Semi-urban MH service network	5	5.4%	0	0.0%	0	0.0%	38	100%	43	100%
Professions (n., %)	Medical professions	35	38.0%	65	58.0%	0	0.0%	8	21.1%	108	100%
	Psychosocial professions	53	57.6%	26	23.2%	69	100%	21	55.3%	169	100%
	General professions	4	4.3%	21	18.8%	0	0.0%	9	23.7%	34	100%
Seniority in profession (Mean, SD)		8.3	10.1	10.4	11.4	9.5	11.4	5.4	7.8	8.9	10.7
Recovery-Oriented Services (Mean, SD) (Rating: 0–7)		4.6	0.6	5.4	0.6	5.2	0.5	4.9	0.6	5.1	0.7
Team Interdependence (Mean, SD) (Rating: 0–21)		12.3	3.0	15.1	2.7	13.3	3.1	13.6	2.9	13.7	3.1
Team Support (Mean, SD) (Rating: 0–7)		4.3	1.1	5.4	0.9	4.5	1.2	5.1	1.1	4.8	1.2
Team Autonomy (Mean, SD) (Rating: 0–7)		4.4	1.3	5.2	1.1	4.8	1.3	5.6	1.1	4.9	1.2
Involvement in the Decision–Making Process (Mean, SD) (Rating: 0–7)		4.3	1.4	5.6	1.0	4.7	1.4	5.6	0.9	5.0	1.3
Work Role Performance (Mean, SD) (Rating: 0–42)		32.9	3.0	36.3	2.8	34.0	3.1	34.9	3.0	34.6	3.2
Conflict Between Co-Workers (Mean, SD) (Rating: 0–21)		10.3	3.7	8.4	2.4	8.9	2.5	7.6	1.5	9.0	2.9
Team Collaboration (Mean, SD) (Rating: 0–28)		16.3	3.1	21.4	3.1	19.2	3.2	20.9	3.6	19.3	3.8
Job Satisfaction (Mean, SD) (Rating: 0–35)		22.5	3.1	26.2	3.3	25.0	3.3	26.0	3.1	24.8	3.6
**Outcomes** **Service user characteristics**		**Category 1** **(n = 84; 25.7%)** ***“Metropolitan network: middle-age men with positive outcomes”***		**Category 2** **(n = 66; 20.1%)** ***“Metropolitan network: older women with few MH problems”***		**Category 3** **(n = 88; 26.9%)** ***“Metropolitan and other networks: service users with complex MH problems and negative outcomes”***		**Category 4** **(n = 89; 27.2%)** ***“Urban and semi-urban networks: young service users with drug disorders”***		**Combined** **(n = 327; 100%)**	

		n/Mean	%/SD			n/Mean	%/SD	n/Mean	%/SD	n/Mean	%/SD

Sites (n., %)	Metropolitan MH service network	84	100%	66	100%	43	48.9%	0	0.0%	193	100%
	Urban MH service network	0	0.0%	0	0.0%	31	35.2%	45	50.6%	76	100%
	Semi-urban MH service network	0	0.0%	0	0.0%	14	15.9%	44	49.4%	58	100%
Gender (n., %)	Female	0	0.0%	66	100%	53	60.3%	45	50.6%	164	100%
	Male	84	100%	0	0.0%	35	39.8%	44	49.4%	163	100%
Age categories (n., %)	18–44	19	22.6%	15	22.7%	28	31.8%	50	56.2%	112	100%
	45–54	36	42.9%	20	30.3%	31	35.2%	20	22.5%	107	100%
	55 and over	29	34.5%	31	47.0%	29	33.0%	19	21.3%	108	100%
Number of MH disorders (Mean, SD)		1.5	0.8	1.4	0.7	2.7	1.0	1.6	1.1	1.8	1.1
Personality disorders (n., %)		0	0.0%	0	0.0%	88	100%	5	5.4%	93	100%
Drug Abuse Screening Test (DAST) (Mean, SD); (rating: 1–5)		2.4	1.7	2.2	1.4	2.4	1.6	3.9	3.9	2.8	2.5
Alcohol Use Disorders Identification Test (AUDIT) (Mean, SD); (rating: 0-10)		6.2	4.8	3.9	3.9	5.6	8.0	4.6	7.3	5.2	6.4
Severity of needs (Mean, SD) (rating: 0–260)		38.9	26.5	39.3	24.5	60.0	33.0	53.4	35.2	48.6	31.7
Adequacy of help received (Mean, SD) (rating: 0–520)		64.6	51.8	51.0	40.2	86.5	52.0	74.8	59.1	70.5	53.2
Alberta Continuity of Services Scale (ACSS) for Mental Health (Mean, SD) (rating: 0–215)		136.3	17.5	131.5	14.5	130.8	16.1	130.6	15.4	132.3	16.1
Recovery Self-Assessment Scale (RSA) (Mean, SD) (rating: 0–410)		168.2	25.1	163.6	17.7	162.3	20.2	164.2	28.0	164.6	23.4
Quality of life (Satisfaction with Life Domains Scale – SLDS) (Mean, SD) (rating: 0–140)		102.0	16.3	99.2	17.8	90.9	20.2	95.3	18.7	96.6	18.8

Four categories emerged across the three service networks for structures, processes and outcomes. A single outcome category: “service users with complex MH problems and negative outcomes” was common to all three MHSN. Network 1, the Metropolitan network, reported two categories for structures (“specialized MH teams”, “primary care MH teams”), two for processes (“senior medical professional”, “psychosocial professionals”), and two other categories for outcomes (“middle-age men with positive outcomes”, “older women with few MH problems”). Networks 2 (Urban) and 3 (Semi-urban) had one category in common for structures (“all teams”), two categories for processes (“psychosocial professionals”: network 2, “all professionals”: networks 3), and also shared a single service user category (“young service users with drug disorders”). The four categories are further described below in relation to the three networks.

### Network 1: The Metropolitan MHSN

This network reflected considerable heterogeneity, with two categories showing opposing characteristics related to structures, processes and service outcomes. In terms of structures, all managers in this MHSN belonged to one of two categories (Table 4). The first category (“Metropolitan network: primary care teams”) utilized relatively fewer clinical approaches, procedures and tools than others. Scores on organizational culture were higher for clan culture than the other cultures; and frequency of interaction with other MH teams and services was lower.

By contrast, category 2 (“Metropolitan network: specialized MH teams”) had relatively high proportions of high ER service users, and used standardized clinical procedures and tools more frequently. Regarding scores on organizational cultures, market culture was highest, and clan culture lowest. Frequency in use of integration strategies was also higher for category 2, whereas frequency of interactions with other MH teams or services was low, as in category 1.

In terms of processes, two categories of MHP in this MHSN produced contrary results on a range of variables: category 1 (“Metropolitan network: psychosocial professionals”) had the lowest scores for all categories on recovery-oriented services, team interdependence, team support, team autonomy, involvement in the decision-making process, work role performance, team collaboration, and job satisfaction, while producing the highest score on conflict between co-workers.

By contrast, category 2 (“senior medical professionals”) had the highest scores on recovery-oriented services, team interdependence, team support, involvement in the decision-making process, work role performance, team collaboration and job satisfaction. Category 2 professionals ranked highest on seniority in profession, and also included MHP from the urban network.

In terms of service user outcomes, all service users in categories 1 and 2 came from this Metropolitan MHSN. Category 1 (“Metropolitan network: middle-age men with positive outcomes”) included exclusively males, 45 to 55 years old, with highest scores on alcohol use disorders, continuity of care, recovery, and QOL, but the lowest score on severity of needs. Category 2 (“older women with few MH problems”) consisted entirely of women, mainly 55 years old and older. This category had the lowest number of MH disorders, including personality disorders, and lowest scores on drug and alcohol use disorders and on adequacy of help received. Category 3 service users (“Metropolitan and other networks: service users with complex MH problems and negative outcomes”) also came mainly from the Metropolitan MHSN, but also included some from the two other MHSN described below. In category 3, 95% of service users had personality disorders, and high levels of other MH disorders; they had the highest scores of all the categories on severity of needs and adequacy of help received, but lowest scores on recovery and QOL, and the second lowest score on continuity of care.

### Network 2: The Urban MHSN

In contrast to the metropolitan MHSN, the urban MSHN was more homogenous. Regarding structures, all managers from this MSHN belonged to category 3 labelled “Urban network: all teams” (Table 4), which registered the lowest proportion of low-income service users. The hierarchical culture was predominant. This category was also notable for the highest frequency of interaction with other teams or services, and lowest score on frequency in use of integration strategies.

In terms of team processes, MHP from the urban MHSN belonged primarily to category 3 labelled “Urban network: psychosocial professionals”. Category 3 had the second lowest scores on team interdependence, team support, team autonomy, involvement in the decision-making process, work role performance, team collaboration, and job satisfaction, but the second to highest score on conflict between co-workers. The remaining MHP were included among the “Metropolitan and Urban networks: senior medical professionals”, previously described.

In terms of service user outcomes, those from the urban MHSN were included in category 3 (Metropolitan and other networks: “service users with complex MH problems and negative outcomes”), described previously, and in category 4, which included the greatest proportion of service users under age 45, with the highest score on drug use disorders. Category 4 also included services users from the Semi-urban MHSN. This category was labelled “Urban and Semi-urban networks: young services users with drug disorders”.

### Network 3: The Semi-urban MHSN

This network was also more homogenous than the metropolitan MHSN. Regarding structures, all managers belonged to category 4 labelled “Semi-urban network: all teams” (Table 4), in which financial resources were most scarce, but managers made the most use of clinical approaches, and had the fewest high ER users. Category 4 also had the highest scores on the entrepreneurial and hierarchical cultures, but the lowest score on frequency of interaction with other MH teams or services.

Regarding processes, almost all professionals in this network belonged to category 4, and seniority in profession was lowest. Category 4 had the highest scores on team autonomy and involvement in the decision-making process, and the lowest score on conflict between co-workers. This category was labelled “Semi-urban network: all professionals”. Most service users from the semi-urban service network were included among “Urban and Semi-urban networks: young services users with drug disorders”, and the others among “Metropolitan and other networks: service users with complex MH problems and negative outcomes”, both of which were previously described.

## Discussion

This study partially confirmed our hypothesis that structures would relate to better team processes, and to more positive outcomes for service users. Although structures seemed to influence team processes in these MHSN, links between team processes and service user outcomes were harder to establish. The establishment of service user categories in terms of: “Metropolitan network: middle-age men with positive outcomes” versus “Metropolitan and other networks: service users with complex MH problems and negative outcomes” demonstrates that service user outcomes were largely associated with clinical characteristics, regardless of network configurations. One explanation may be that structures and processes were quite distal to outcomes [63]. According to Kilbourne et al. [63], as compared with other health sectors, quality assessments in MH care tend to be weaker due to multiple structural barriers such as lack of professional training and support and cultural obstacles to integrated care, contributing to poor outcomes. Moreover, a recent review on outcomes stemming from multidisciplinary collaboration in primary health care found that the relationship between processes and outcomes was difficult to determine and, contrary to investigations on structures, processes were often poorly described in studies [64]. In Quebec, the 2005 MH Action Plan also provided few descriptions of the operational mechanisms (processes) underlining the reform, as compared with descriptions of new structures and services and their implementation, which were fully described [65].

The heterogeneity revealed within the metropolitan MHSN, which brought together two categories with very different characteristics in terms of structures and processes, was an interesting finding. Some studies also found that integrated care was more difficult to implement in large MHSN, especially in those with a psychiatric hospital, as was the case for the Metropolitan MHSN in this study. This type of networks tended to operate in silo [66,67,68]. Concerning structures, the results for category 1 within the metropolitan MHSN described the situation of new MH primary care teams that were created in HSSC over the course of the MH reform; whereas category 2 reflected that of specialized MH teams. The capacity of primary care services to assess and treat MH disorders (MHD) is frequently viewed as limited [69,70,71,72], which may explain the less frequent use of clinical procedures, tools, and approaches among “Metropolitan network: primary care teams” (category 1). The clan culture, which links MHP to family members while focusing less on standardization and best practices, is viewed as typical of primary care teams [42]. By contrast, “Metropolitan network: specialized MH teams” (category 2) reflected a better mix of organizational cultures, and possessed strengths emanating from each: stability and efficiency (hierarchical culture); competiveness and inter-organizational interaction (market culture); flexibility, innovation and external focus (entrepreneurial culture) [73]. According to Tietschert et al. [22], a good mix of organizational cultures is desirable to produce a higher patient-perceived level of integrated care.

Concerning structures, the exclusive distribution of managers from the Semi-urban MHSN within a single category (category 4) reflected the concentration of both primary care and specialized MH teams within a single institution, enhancing a shared vision and practices while promoting integrated care. The entrepreneurial culture, more predominant in the Semi-urban MHSN, suggested better adaptive ability among MH teams [73]. Tiestchert et al. [22] found that the entrepreneurial culture was strongly associated with a high level of patient-perceived integrated care. The concentration of all managers from the Urban MHSN within a single category (category 3) is more difficult to explain, since primary care teams were located in a HSSC and specialized services in a MH university institute, similar to the Metropolitan MHSN. It is possible that the primary care and specialized MH teams had successfully created an integrated vision and practices, which are usually reported as positively associated with better quality and continuity of care [32]. The accumulation of high ER users in the Urban MHSN might explain the frequent use of clinical procedures, tools and approaches. The highest frequency of interaction with other teams or services in the Urban MHSN combined with the lowest scores on use of integration strategies in this network seemed contradictory. One explanation may be that the long-term collaboration among MHP from different teams and organizations in the Urban MHSN had eliminated the need to formalize these arrangements. Formalized integration mechanisms are more pressing when few traditions around collaboration exist, and would be needed to insure collaboration in a reform context [74].

Concerning team processes, the concentration of high scores among the Metropolitan and Urban networks: senior medical professionals” (category 2) and low scores among “Metropolitan network: psychosocial professionals” (category 1) was logical, as these variables are often strongly related even though they measure distinct concepts. Previous studies have identified associations between job satisfaction and team collaboration [75], involvement in the decision-making process [76], team autonomy [77], recovery-oriented services [78] and lower conflict between co-workers [76]. Associations were also found between team interdependence, team support and work role performance [17,79].

The low team process scores identified among “Metropolitan network: psychosocial professionals” suggests that these professionals, who were transferred from specialized MH services to the newly formed primary care teams in this network, may have experienced adaptation problems in their new environment. According to a recent literature review, professionals involved in joint practice often have difficulties in adapting their usual work methods [80]. Better overall team process scores were found instead among the “Metropolitan and Urban networks: senior medical professionals”, which was also the only category with mainly medical professionals. Studies have identified greater satisfaction with teamwork among physicians as compared with professionals lower down in the team hierarchy (e.g., nurses, social workers), who are more likely to burn out or quit their jobs [75,81,82,83]. Moreover, the hierarchical culture predominated in “Metropolitan network: specialized MH teams”, suggesting clear role definition in these teams, as well as stability, and greater capacity among team members to control their jobs [84] and work effectively. Experienced professionals would also enjoy mutual familiarity, another facilitator of positive team processes (e.g., team collaboration, team interdependence, job satisfaction, and less team conflict) [85].

Concerning processes, almost all professionals from the Semi-urban MHSN were concentred in a single category (category 4), and had the least seniority. Younger professionals, who tend to be more dynamic and open to innovation, characteristic of the entrepreneurial culture [42], were predominant in this network. High scores on team autonomy and involvement in the decision-making process for the Semi-urban MHSN may also have reflected their working conditions in a remote area with relatively few resources [86]. A more proactive and autonomous approach is another feature associated with the entrepreneurial culture. Finally, the profile for MHP in the “Urban network: psychosocial professionals” was quite similar to that for “Metropolitan network: psychosocial professionals”, which may explain their low team process scores.

Concerning service user outcomes, higher QOL and recovery scores in the “Metropolitan network: middle-age men with positive outcomes” (category 1) likely resulted from the greater availability and continuity of services in this network. Strong associations between quality of life, recovery and continuity of care were previously identified [57,58,87]. The substantial financial resources available to the Metropolitan MHSN would also have allowed for better follow-up of “Metropolitan network: middle-age men with positive outcomes”, who were mainly affected by alcohol use disorders requiring ongoing and coordinated help from various resources such as MH services, SUD rehabilitation centers, primary care services, and support groups like Alcoholics Anonymous.

Outcomes were also positive for the “Metropolitan network: older women with few MH problems” (category 2), except on adequacy of help received. Service users with less severe conditions may have been disadvantaged, e.g., in terms of wait times for services, as complex cases usually receive priority care. Moreover, MH needs tended to be unmet among older service users [88,89], particularly those related to co-occurring MH and physical health disorders [88].

The widespread negative outcomes described for “Metropolitan and other networks: service users with complex MH problems and negative outcomes” (category 3) seem to reflect the complexity of MH profiles in this category, including personality disorders and higher risk of self-harm or suicide ideation, which presented challenges for both primary care and specialized MH service teams [90]. The integration of several sectors (including health and social services, but also employment and education) is often necessary in order to meet the needs of this vulnerable population [80]. Service users with personality disorders were also high users of ER and other services and were often dissatisfied with the adequacy of help received [91]. Severity of needs, previously identified as associated with lower QOL [92,93,94,95] and lower recovery [96,97,98,99], was highest among “Metropolitan and other networks: service users with complex MH problems and negative outcomes”, even though they reported greater adequacy of help received. These results seem contradictory, but may have been offset by the tendency of MH services to prioritize treatment and follow-up for service users with more severe, complex or co-occurring disorders [100,101,102].

Finally, results identified “Urban and Semi-urban networks: young service users with drug disorders” (category 4). According to previous research, these service users are more likely to drop out prematurely from services [103], which may explain their lower service continuity scores in this study. Another characteristic of this group is their greater reliance on self-help rather than help-seeking [104]. Interestingly, service users from the Semi-urban MHSN category tended to be followed by MHP with lower professional seniority (team processes). Yet these professionals, being younger, may have felt more affinity with younger service users, possibly facilitating professional-service user relationships.

## Limitations

This study had limitations that should be addressed. First, cluster analyses necessarily use a limited number of variables. Second, as most service users used multiple services, it was not possible to clearly associate each service user group with a specific network or category in terms of structures and processes. Third, the use of cross-sectional data precluded an interpretation of results for the four categories over time. Finally, major reforms, as in the case of Quebec, take time to implement fully and for positive outcomes to become apparent.

## Conclusions

This study was innovative in attempting to identify categories of related variables associated with structures, team processes and services user outcomes in three Quebec MHSN using cluster analyses. The main contribution of the study was to identify service user outcomes that were largely associated with clinical characteristics, regardless of network configurations with different structures and team processes. All networks included service users with complex MH profiles, including multiple MHD such as personality disorders, which contributed to negative outcomes. Another important finding of this study was the greater heterogeneity identified in the Metropolitan MHSN in terms of structures and team processes, as compared with the Urban and Semi-urban MHSN, which suggests that implementation of the MH reform was relatively more difficult in this type of network. More particularly, primary care teams in the Metropolitan MHSN, which mainly consisted of psychosocial professionals, made less use of clinical procedures, tools, and approaches than did specialized MH teams, which may have affected their capacity to evaluate and treat service users with MHD. The results also suggested that a good balance of organizational cultures was associated with better team processes. This study also revealed that more positive team processes were associated with greater presence of medical and senior professionals among team members, which was more characteristic of specialized MH service teams.

Overall, this study points to the need for better support to psychosocial professionals on the part of MH decision makers. Shared-care initiatives should be promoted, and additional resources allocated to reinforce MH services in primary care, including follow-up of younger service users with SUD, and most especially high ER users with complex clinical profiles. Extended implementation of best-practices and integration strategies in all service networks, especially in primary care teams, positively influenced team processes scores, and other outcomes, which implies that these measures should be promoted. Finally, this study supports greater promotion of organizational cultures focused on innovation and results-orientation, as well as greater inter-organizational interaction.
